# Mapping motor exercise interventions in Parkinson's disease: a scoping review of intervention types, outcomes, adherence, and dropout

**DOI:** 10.3389/fspor.2026.1881313

**Published:** 2026-08-19

**Authors:** G. D’Alessandro, A. D’Ermo, T. Di Libero, A. Rodio, L. Falese, F. Stocchi, M. Goffredo, E. Padua, B. Tosti, M. F. De Pandis

**Affiliations:** 1 Department of Human, Social and Health Sciences, University of Cassino and Southern Lazio, Sustainable Living Concept Laboratory “Marco Marchetti” (XLab), Campus Folcara, Via S. Angelo, Cassino, Italy; 2 IRCCS San Raffaele Roma, Cassino, Italy; 3 European University of Technology Eut+, European Union; 4 IRCCS San Raffaele Roma, Rome, Italy; 5 Department of Human Science and Promotion of Quality of Life, San Raffaele Rome Open University, Rome, Italy

**Keywords:** adherence, dropout, exercise rehabilitation, Parkinson's disease, physical exercise, tailored training

## Abstract

**Objective:**

This scoping review aimed to map the current evidence on exercise interventions for people with Parkinson's disease (PD), describing the most frequently investigated intervention types and synthesizing the evidence on motor and non-motor outcomes, as well as adherence and dropout patterns.

**Methods:**

This scoping review was conducted in accordance with the PRISMA-ScR guidelines and used an umbrella evidence-mapping approach. A systematic search of PubMed/MEDLINE, Embase, Web of Science, Cochrane Library, and Scopus was performed for studies published between January 2010 and February 2026. Eligible sources included systematic reviews, meta-analyses, network meta-analyses, and a limited number of primary studies to complement areas where synthesized evidence was limited. Two independent reviewers screened the studies and extracted data on intervention character-istics, exercise dose (FITT parameters), motor and non-motor outcomes, adherence, and dropout.

**Results:**

Twenty-six studies met the eligibility criteria. Considerable heterogeneity was observed across intervention characteristics, exercise dose, and outcome measures. Overall, different exercise modalities were associated with improvements in specific motor and non-motor outcomes, although the evidence varied among the included evidence syntheses. Adherence was generally higher in supervised and socially engaging interventions, whereas dropout was mainly associated with disease-related, logistical, technological, and psychosocial factors.

**Conclusions:**

The available evidence suggests that different exercise modalities may provide complementary benefits on motor and non-motor outcomes, supporting individualized exercise prescription according to patients’ clinical characteristics, rehabilitation goals, disease stage, and feasibility. Future research should prioritize greater standardization of exercise protocols and more consistent reporting of exercise dose, adherence, and dropout to facilitate comparisons across studies and strengthen the evidence base.

## 1 Introduction

Parkinson's disease (PD) is a progressive and complex neuro-degenerative disorder that predominantly affects older adults, particularly those over 60 years of age (1), and is recognized as the second most prevalent neuro-degenerative disease worldwide (2). The prevalence of PD is rapidly increasing, with projections indicating a doubling of cases globally, from approximately 6.2 million to more than 12 million by 2040 (3). The core symptoms of PD primarily involve dysfunction of the somatomotor system, manifesting as rigidity, bradykinesia, tremor, gait dysfunction, and postural instability (1, 4). The progression of these symptoms compromises patients' ability to perform activities of daily living and reduces independence (5). Moreover, postural instability increases the risk of falls, a problem affecting more than 50% of patients (5, 6). In addition to motor impairments, the disease is often accompanied by non-motor symptoms, including cognitive decline, sleep disturbances, anxiety, and depression (7, 8).

The primary treatment for PD remains pharmacological intervention, such as levodopa, and, in selected cases, surgery, including deep brain stimulation (7). However, the effectiveness of pharmacotherapy is time-limited, may require frequent dose adjustments in advanced stages, and can lead to side effects such as dyskinesias or reduced balance and walking ability (3, 5). To address these limitations, interest in complementary inter-ventions, particularly physical exercise, has grown substantially (7). Physical exercise is increasingly recognized not only as a means of reducing functional limitations but also for its potential benefits on neuro-plasticity and the self-repair capacity of the central nervous system (4). Physical activity has also been described as a low-cost, potentially neuro-protective strategy associated with a reduced risk of developing PD and with the promotion of brain plasticity through neurotrophic mechanisms (9). Thus, physical exercise should not be considered merely a supportive activity, but rather a fundamental therapeutic strategy for PD management, acting synergistically with pharmacological treatment (8, 10).

Despite broad scientific consensus on the overall benefits of exercise, significant uncertainty remains regarding which type of exercise may be most appropriate for specific outcomes (3, 7, 11). In recent years, research has explored a wide range of motor strategies, including aerobic exercise, such as walking, cycling, and treadmill training with or without body-weight support; strength training; and multi-component programs combining aerobic, resistance, balance, and gait training to simultaneously target multiple functional and physiological systems (1, 12). Balance-oriented interventions include Tai Chi, Qigong, and dance-based approaches such as tango (13). Other investigated approaches include exergaming and virtual reality, external cueing, dual-task training, LSVT-BIG, robotic technologies for gait rehabilitation, and water-based therapies such as hydrotherapy (14–16). Additional technology-supported and innovative approaches have also been described in recent literature.

There are relatively few studies directly comparing multiple exercise modalities, and the available literature often addresses different outcomes or uses substantially different protocols (17). In addition, dropout deserves specific attention in studies on physical exercise in PD because it is central both for evaluating intervention acceptability and as a potential source of methodological bias, as highlighted in recent literature (8). While many reviews focus primarily on intervention efficacy, less attention has been devoted to adherence patterns, reasons for withdrawal, and factors influencing long-term participa-tion.

Therefore, the aim of the study was to identify the main types of exercise interventions studied in Parkinson's disease and to examine the distribution of intervention categories within broader evidence syntheses, summarizing their effects on motor and non-motor outcomes, highlighting the importance of adherence and dropout rates.

## 2 Materials and methods

The protocol for this scoping review was not publicly registered; however, the methodological steps strictly followed the PRISMA-ScR guidelines.

Although this review followed the PRISMA-ScR framework, it adopted an umbrella evidence-mapping approach by primarily synthesizing evidence from systematic reviews and meta-analyses while incorporating selected primary studies when they provided unique or complementary information not available in secondary evidence syntheses.

The literature search was performed in PubMed, MEDLINE, Embase, Web of Science, Cochrane Library, and Scopus, with coverage from January 2010 to February 2026. Keywords used included Parkinson, physical exercise interventions, rehabilitation, motor reconditioning, physical activity, movement therapy, aerobic training, resistance training, balance training, gait training, videogame, exergame, aquatic, cueing, dual task and dropout. These keywords were used both as single terms and in combination.

The following string of terms was used: [“Parkinson's disease”(Mesh) OR “Parkinson*”] AND (“physical exercise”[All Fields] OR “rehabilitation”[All Fields] OR “motor recondi-tioning”[All Fields] OR “aerobic training”[All Fields] OR “resistance training”[All Fields] OR “balance training”[All Fields] OR “gait training”[All Fields] OR “exergame”[All Fields] OR “aquatic’[All Fields] OR “dropout”[All Fields]).

### 2.1 Eligibility criteria

Eligible sources included systematic reviews, meta-analyses, network meta-analyses, and selected primary studies. Primary studies were selectively included to complement evidence from meta-analyses in areas where synthesized evidence was limited or absent, in order to comprehensively map both synthesized and primary evidence on exercise-based interventions in PD. Additional filters included English language, adult population, and publication period from 2010 to 2026. Studies were excluded if they did not include patients diagnosed with PD, involved interventions not attributable to structured motor strategies or exercise, or consisted of uncontrolled case reports. This scoping review adopts an umbrella approach prioritizing evidence from meta-analyses, complemented by selected primary studies where necessary.

### 2.2 Study-selection

Two independent reviewers (G.D.A. and B.T.) screened the titles and abstracts and evaluated the full texts using the Rayyan review management software. Any disagreements were resolved through discussion or consultation with a third reviewer (A.R.).

### 2.3 Data extraction

Data were extracted by two independent reviewers using a standardized form developed by the research team and preliminarily tested on a sample of studies to verify their clarity and completeness. It was not necessary to contact the authors of the included studies. For each included study, the following data were extracted: sample size, mean age, disease stage according to the Hoehn and Yahr scale, type of intervention, exercise dose in terms of volume, intensity, frequency, and duration, and clinical outcomes, both motor and non-motor. These outcomes included validated measures such as the Movement Disorder Society–Unified Parkinson's Disease Rating Scale, Part III Motor Examination (MDS-UPDRS III) (18), Timed Up and Go (TUG) (19), Berg Balance Scale (BBS) (20), 6-Minute Walk Test (6MWT) (21), Parkinson's Disease Questionnaire–39 items (PDQ-39) (22), Freezing of Gait Questionnaire (FOG-Q) (23), and Mini-BESTest (Mini-Balance Evaluation Systems Test) (24). Non-motor outcomes included health-related quality of life, depression, anxiety, cognition, sleep, fatigue, and other non-motor symptoms, as assessed by validated instruments reported in the included reviews (e.g., NMSS/NMSQ, BDI, HAM-D, GDS, HRQoL, MMSE, MoCA, and FSS).

When available, quantitative effect estimates and intervention dose parameters were extracted as reported in the original studies or reviews, without performing additional quantitative synthesis. Information on the certainty of evidence was recorded when explicitly reported in the included meta-analyses and network meta-analyses. No independent assessment of evidence certainty was performed by the review authors, in line with the scoping review methodology. For individual primary studies, methodological aspects such as randomization, blinding, and definition of primary outcomes were considered descriptively, without deriving an aggregated GRADE (Grading of Recommendations Assessment, Development and Evaluation) classification, due to the high heterogeneity of interventions.

## 3 Results

**Figure 1:**
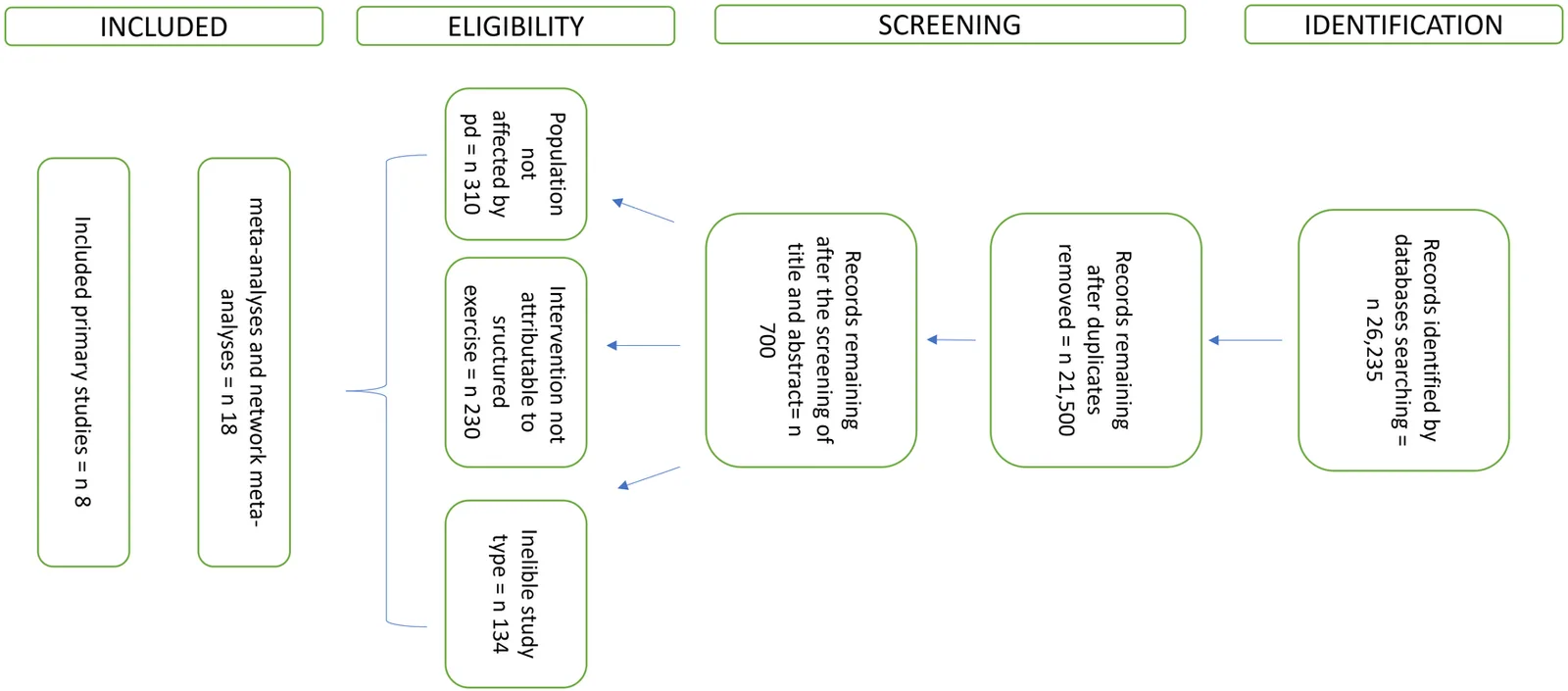
Study selection process flow diagram.

The search process, illustrated in Figure 1, started with 26,235 records gathered from electronic databases and other sources. After removing duplicates, we screened 21,500 records by title and abstract. Of these, 700 articles were reviewed in full, and ultimately, 26 studies were included in the final synthesis, 18 of which were meta-analyses or network meta-analyses, while the remaining 8 were primary studies. Across the included literature, substantial heterogeneity emerged in intervention characteristics, including exercise modality, intensity, duration, supervision level, and outcome selection. Despite this variability, consistent outcome-specific associations between exercise categories and clinical domains were identifiable across studies, allowing the identification of recurring intervention patterns for motor symptoms, balance and mobility, gait performance, non-motor outcomes, and participant retention.

It be noted that a substantial proportion of the evidence synthesized in this review originates from comprehensive systematic reviews, meta-analyses (MAs), and network meta-analyses (NMAs). Due to this umbrella overview design, an absolute manual recalculation of primary study frequencies across all included sources was methodologically unfeasible. Many of the high-level reviews share a significant degree of overlap, capturing the same landmark primary randomized controlled trials (RCTs). Attempting an unadjusted pool of individual study frequencies would have led to severe double-counting and skewed prevalence data. Consequently, to map out the most frequently investigated motor exercise strategies accurately without compounding data duplication, our synthesis prioritizes tracking the intervention categorization and structural distribution directly from the large-scale pooled reviews themselves.

Specifically, trends regarding frequency were extrapolated from the most comprehensive and methodologically sound evidence syntheses available, such as the Cochrane network meta-analysis by Ernst et al., which alone includes 156 randomized controlled trials. This strategy provides a comprehensive overview of the relative representation of different exercise modalities (e.g., multi-component training, dance, aerobic exercise, resistance training, and treadmill training) in the literature on Parkinson's disease. The motor strategies are summarized in Table 1.

**Table 1:** Physical exercise intervention sources included in the synthesis.

Activity in source	Focused article title	Disease stage (H&Y)	Main findings	FITT parameters	Authors
Combined Exercise & Self-Management	A physiotherapy group exercise and self-management approach to improve physical activity in people with mild-to-moderate Parkinson’s disease: a randomized controlled trial	Mild–Moderate	Protocol focusing on increasing daily steps and functional gait through behavioral change.	F: RCT protocol; I: combined; T: NR; Type: functional physiotherapy.	(46)
Aerobic Exercise	A systematic review and meta-analysis on effects of aerobic exercise in people with Parkinson’s disease	I–III	Significant improvements in UPDRS-III, TUG, and 6MWT; aerobic exercise is effective for global motor function.	F: 2-5 sessions/week; I: 40%–80% HRmax; T: 20–60 min; Type: tread-mill/cycling.	(5)
Mind–Body Exercise	Are movement-based mindful exercises, including Qigong, Tai Chi, and Yoga, beneficial for stroke and Parkinson’s disease? A scoping review	I–IV	Positive effects on postural balance and motor symptoms; mind–body practices are generally well tolerated.	F: 1-4 sessions/week; I: low; T: 30–90 min; Type: postural and mindful movement.	(34)
Twenty-Four Exercise Modalities	Comparative efficacy of 24 exercise types in Parkinson’s disease	2.0–3.1	Network analysis showing dance and multidisciplinary programs as top-ranked interventions for balance.	F: 2-5 sessions/week; I: varied; T: 4–24 weeks; Type: comparative exercise modalities.	(14)
Aerobic vs. Resistance Training	Effectiveness of aerobic and resistance training on motor symptoms in Parkinson’s disease: a systematic review and network meta-analysis	I–III	Short-period high-intensity resistance training was identified as the most effective strategy for motor symptom reduction.	F: 2-3 sessions/week; I: high; T: *<* 6 to *>* 12 weeks; Type: aerobic vs. resistance training.	(28)
Resistance Training	Effects of resistance training on measures of muscular strength in people with Parkinson’s disease: a systematic review and meta-analysis	Mean 2.0	Resistance training significantly in-creased leg press and knee-extension strength compared with control conditions.	F: 2-3 sessions/week; I: 50%–80% 1RM; T: 30–60 min; Type: progressive resistance exercise.	(32)
Ten Exercise Interventions	Effects of ten different exercise interventions on motor function in Parkinson’s disease patients: a network meta-analysis of randomized controlled trials	I–III	Dance ranked first for balance, whereas virtual reality/exergaming was most effective for functional mobility.	F: 2-3 sessions/week; I: RPE 12–15; T: 15–60 min; Type: comparative network meta-analysis.	(11)
Sustaining Motor Function	Exercise sustains motor function in Parkinson’s disease: evidence from 109 randomized controlled trials on over 4,600 patients	I–III	Nordic walking showed the highest probability of improving mobility performance.	F: mean 2.5 sessions/week; I: varied; T: mean 14.5 weeks; Type: comprehensive exercise interventions.	(37)
Exergaming	Exergaming compared with conventional physical exercise interventions on health status in older people with Parkinson’s disease	I–III	Interactive video games significantly improved balance and quality of life.	F: 2-3 sessions/week; I: RPE 15; T: 15–60 min; Type: Kinect/Wii Fit.	(35)
Online Functional Program	Feasibility and preliminary efficacy of an online home-based functional exercise program for Parkinson’s disease: a pilot study	1.9-2.0	High compliance with elastic-band and step-box exercises; improvements in motor and non-motor signs.	F: 2 sessions/week; I: functional high intensity; T: 60 min, 8 weeks; Type: remote Zoom-based training.	(38)
Functional Rehabilitation	Functional physical rehabilitation and self-assessment of physical activity in Parkinson’s disease	NR	Professional supervision improved accuracy of exercise execution; rehabilitation increased daily physical activity volume.	F: daily physical activity assessment; I: progressive; T: long-term; Type: self-assessment and supervised rehabilitation.	(27)
Dose and Balance	Optimal dose and type of exercise improve overall balance in adults with Parkinson’s disease: a systematic review and Bayesian network meta-analysis	Mean 2.3	Balance/gait training and multi-component programs were identified as optimal strategies for balance stability.	F: 2-3 sessions/week; I: 60-min sessions; T: 8–12 weeks; Type: dose–response exercise analysis.	(26)
Motor Intervention Patterns	Parkinson’s disease motor intervention patterns: a network meta-analysis based on patient motor function	I–III	Dance ranked first for improving 6MWT distance; findings support the need for varied interventions.	F: 1-5 sessions/week; I: 15-120 min; T: 4-96 weeks; Type: motor-pattern analysis.	(2)
General Physical Exercise	Physical exercise for people with Parkinson’s disease: a systematic review and network meta-analysis	I–III	Cochrane review showing that dance and gait/balance training were associated with favorable motor outcomes.	F: 1-7 sessions/week; I: mean 67 min; T: mean 4-12 weeks; Type: Cochrane network meta-analysis.	(8)
Exergames	Exergames in neurocognitive disease management in elderly: a narrative review of therapeutic benefits and applications	I–III	Exergames are a viable alternative to traditional exercise, significantly improving cognitive functions, balance, and walking ability in elderly individuals with neurocognitive disorders.	F: 2 to 5 sessions/week; I: moderate to vigorous; T: from 4 to 24 weeks s; Type: video gaming often with dual-task training.	(16)
Aerobic Exercise and Quality of Life	The effects of aerobic exercise on quality of life and motor function in patients with Parkinson’s disease: a systematic review and meta-analysis	I–III	Aerobic exercise significantly improved BBS and overall quality-of-life scores.	F: 3-4 sessions/week; I: 60%-80% HRR; T: 30-60 min; Type: aerobic exercise.	(43)
Wuqinxi Qigong	The feasibility and positive effects of Wuqinxi exercise on cognitive and motor functions: a pilot study	I–III	Traditional animal-imitation exercises improved attention, memory, and motor function.	F: 2-5 sessions/week; I: comfortable; T: 90 min; Type: traditional Qigong.	(30)
Home-Based Exercise	The effect of home-based exercise on motor symptoms, quality of life, and functional performance: a systematic review and meta-analysis	II–III	Remotely supervised exercise was safe and effective; more than three weekly sessions may provide greater motor gains.	F: 2-7 sessions/week; I: 30-60 min; T: 4-24 weeks; Type: home-based exercise.	(33)
20 Exercise Types	Effectiveness of different exercises in improving postural balance among Parkinson’s disease patients: a systematic review and network meta-analysis	I–III	Exergaming best for TUG; Dance best for BBS; Rhythmical Auditory best for Mini-BESTest.	F: 2.72 sessions/week; I: moderate to vigorous; T: 51.78 min for session; Type: multimodal.	(25)
Treadmill Training	Treadmill training for patients with Parkinson’s disease	I–III	Improved gait speed (0.09 m/s) and stride length; small to moderate clinical relevance.	F: 3-4 sessions/week; I: gradually increasing speed; T: 20–60 min; Type: treadmill.	(51)
Home-based Exercise	Home-based prescribed exercise improves balance-related activities in people with Parkinson’s disease and has benefits similar to center-based exercise: a systematic review	I–III	Improved balance and gait speed; benefits similar to equivalent center-based exercise.	F: varied; I: 150 min/week; T: 6 weeks; Type: Prescribed (Gait, balance, strength).	(39)
Dance Therapy	Dance therapy for Parkinson’s disease: a systematic review	I–III	Improved tremor, bradykinesia, and balance; Argentine Tango ranked most effective.	F: 2-7 sessions/week; I: Vigorous/Moderate; T: 1 h sessions (6–12 weeks); Type: Dance (Tango, Irish, Ballet).	(13)
Multi-modal PA	The Impact of Physical Activity on Non-Motor Symptoms in Parkinson’s Disease: A Systematic Review	ND	Significant positive impact on global non-motor symptoms: depression, apathy, fatigue, sleep, and cognition.	F: 2 - 4 sessions/week; I: Varied; T: 20–90 min/session; Type: Aerobic, Resistance, Mind-Body.	(52)
Prog. Resistance Training	Progressive resistance training in Parkinson’s disease: a systematic review and meta-analysis	I–III	No evidence of superiority over other physical training for walking speed, distance, or TUG.	F: 2-3 sessions/week; I: progressive; T: 1.5 - 24 months; Type: resistance and strength.	(31)
Community-based Physical Activity	Motivators and Barriers Affecting Exercise in Patients With Parkinson’s Disease	I–III	17% of patients are inactive; non-motor symptoms (fatigue/apathy) are primary barriers, while intrinsic motivation drives high-exercise groups.	F: self reported; I: varied; T: general PA; Type: Walking, pilates, weights.	(12)
Telemedicine (e-Exercise)	Effectiveness of Telemedicine Interventions on Motor and Non motor Outcomes in Parkinson Disease: Systematic Review and Network Meta-Analysis	I–III	e-Exercise is the most effective remote strategy for total motor symptoms; e-Cognitive interventions best for depression and anxiety.	F: 3 sessions/week; I: moderate to high; T: 30-60 min; Type: aerobic and functional.	(29)
Comprehensive Physiotherapy	Physiotherapy in Parkinson’s Disease	I–III	Meta-analysis confirms significant motor benefits (MDS-UPDRS III), balance (BBS), and gait improvements across various modalities.	F: 5 sessions/week; I: moderate to high; T: 30-90 min; Type: Conventional PT, Resistance, Treadmill, Dance.	(1)

### 3.1 Motor exercise strategies most frequently investigated

Walking is consistently reported as one of the most frequently prescribed physical activities for people with PD (12). More broadly, the literature most commonly investigated aerobic training, resistance training, balance and gait-oriented rehabilitation, mind–body interventions, and dance-based programs. These intervention categories reflect both traditional physiotherapy approaches and more recent strategies integrating coordination, attentional engagement, and externally paced movement. According to the Cochrane review by Ernst et al. (2023) (8), which included 156 studies involving 7,939 participants, multi-domain programs (60 studies) and balance, gait, and functional training protocols (58 studies) were the most frequently represented intervention categories. Mind–body interventions, such as Tai Chi, yoga, and Qigong, accounted for 23 studies, followed by endurance training (20 studies), strength training (17 studies), and dance-based approaches (13 studies).

This distribution suggests that interventions targeting multiple functional domains simultaneously are particularly prominent in the literature, likely reflecting the multi-factorial nature of motor impairment in PD. At the same time, the consistent presence of endurance, strength, and coordinative movement-based interventions highlights the absence of a single dominant therapeutic model, with different strategies being investigated according to specific clinical targets and rehabilitation settings.

This pattern is consistent across the broader body of evidence published over the past decade. Beyond the findings of the Cochrane review (8), several systematic reviews and meta-analyses confirm that multi-component exercise programs, together with strength-and balance-oriented interventions, are the most extensively investigated rehabilitation strategies for Parkinson's disease (1, 14, 25, 26). This consistency across the literature reinforces the view that the complexity of Parkinson's disease is best addressed through multimodal exercise interventions targeting multiple functional domains simultaneously.

### 3.2 Reported effects of exercise interventions

#### 3.2.1 Overall motor symptoms

Motor symptoms were primarily assessed using UPDRS-III or MDS-UPDRS III scores. Across studies, improvements in global motor performance were reported for several intervention categories, although the magnitude of effects varied depending on training characteristics and outcome definitions. Network meta-analyses identified power training as the intervention associated with the largest effect sizes on global motor symptoms, followed by yoga, body-weight-supported treadmill training, and resistance training (7). Furthermore, structured interventions like functional physical rehabilitation appear cru-cial not only for motor gains but also for improving patients' self-awareness; indeed, participation in such programs significantly enhances the accuracy of self-reported physical activity, reducing the systematic overestimation often observed in this population (27).

Improvements following high-intensity aerobic and resistance exercise were also reported (28), suggesting that interventions providing sufficient neuromuscular and cardiovascular stimulus may contribute to clinically meaningful motor improvements. Additional approaches explored in recent studies included archery-based exercise and telemedicine-delivered training programs (29), reflecting increasing interest in accessible and task-oriented intervention formats. Coordinative mind–body practices, such as Wuqinxi, were associated with improvements in both UPDRS-III and TUG scores (30), indicating potential benefits that extend from global motor symptoms to functional mobility.

Long-term progressive resistance exercise produced sustained improvements over 24 months compared with non-progressive protocols (31), supporting the relevance of structured progression for maintaining treatment effects over time. Overall, improvements in global motor symptoms were observed across multiple exercise modalities, with particularly consistent findings emerging for interventions combining progressive overload, coordinated movement practice, and moderate-to-high training intensity (32).

Overall, findings from recent systematic reviews are highly consistent, confirming that exercise-based rehabilitation improves global motor symptoms, most commonly assessed using the MDS-UPDRS III, with the greatest benefits generally observed after progressive, moderate-to-high intensity, and multimodal exercise interventions (1, 5, 33).

### 3.3 Balance and functional mobility

Balance and functional mobility outcomes were commonly assessed using BBS, TUG, and Mini-BESTest. Across studies, several intervention categories demonstrated favourable effects on different components of postural control and mobility performance. According to the network meta-analysis by Wang et al. dance achieved the highest SUCRA value for BBS (81.3%) among the exercise modalities evaluated, suggesting a particularly strong association with improvements in balance performance (25). In the network meta-analysis by Qian et al. aquatic exercise ranked highest for steady-state static balance (14)., while Tai Chi demonstrated consistent efficacy across multiple analyses (2, 34), supporting the relevance of interventions involving controlled weight shifting and multidirectional movement. Pilates and body-weight-supported treadmill training were associated with improvements in dynamic balance (14).

Exergaming interventions were frequently ranked among the most effective approaches for improving TUG performance (16, 25, 35), suggesting that interactive environments combining motor execution with real-time feedback may facilitate mobility-related adaptations. Rhythmic auditory exercise demonstrated strong effects on Mini-BESTest outcomes (25), highlighting the role of externally paced timing cues in improving locomotor coordination.

Aerobic exercise interventions were also associated with improvements across TUG, BBS, and UPDRS-III scores (5), indicating that endurance-oriented training may support multidimensional functional gains. Recent evidence also highlights a dose response relationship between exercise and balance, with dance and aerobic training showing optimal effects at specific weekly volumes (26).

Community-based exercise programs further demonstrated improvements in walking capacity and balance outcomes (36), while longitudinal analyses showed progressive improvement in TUG performance over time (37). Together, these findings indicate that interventions incorporating rhythmic stimulation, supported gait practice, or multidirectional postural control are consistently associated with improvements in balance and functional mobility (36)

Overall, evidence from multiple network meta-analyses consistently indicates that task-specific interventions, particularly dance-based exercise, exergaming, and rhythmic auditory stimulation, achieve the greatest improvements in balance and functional mobility outcomes, although rankings vary according to the specific outcome measure and analytical approach (11, 14, 25). Conventional aerobic exercise and structured physio-therapy also provide significant functional benefits, while recent evidence suggests that remotely supervised home-based programs may achieve mobility outcomes comparable to center-based rehabilitation (1, 33, 38).

### 3.4 Walking distance and speed

Endurance, functional cardiovascular capacity, and prolonged walking tolerance were mapped through the 6MWT. Dance Therapy: When examining walking capacity and total distance covered, comprehensive motor pattern, the network meta-analysis by Zhao et al. (2) ranked dance interventions highest for improving 6MWT distance. Aerobic and Resistance Interventions: Standard aerobic training (5) yielded reliable increases in submaximal cardiovascular endurance. Conversely, progressive resistance training (31) successfully built localized force but demonstrated limited standalone superiority in ex-panding total 6MWT distance compared to integrated active controls.

Spatiotemporal gait metrics were examined using specialized walk tests and the FOG-

Q. Treadmill Training: Systematic reviews dedicated to mechanized gait training (39) reported precise improvements in raw gait speed (with a mean clinical increase of 0.09 m/s) and a proportional expansion of stride length. Nordic Walking: The network meta-analysis by Zhang et al. (37) reported that Nordic Walking had the highest probability of improving long-term gait performance.

### 3.5 Non-motor symptoms and quality of life

Health-related quality of life (HRQoL) was mapped almost exclusively using the validated PDQ-39 summary index. Multi-Modal and Mind-Body Interventions: Comprehensive physical activity reviews (40) and mindfulness-based interventions (34) proved highly effective at lowering total PDQ-39 scores, indicating that holistic, socially engaging, and multi-modal training strongly addresses the multi-faceted nature of the disease. Exergaming and Home Prescribed Exercises: Interactive digital platforms (35) and structured home-based protocols (41) showed significant post-intervention enhancements in HRQoL, reflecting that high user enjoyment (in games) and the convenience of remote training (at home) directly translate to better self-reported health status. (see Table 2 for a detailed summary of clinical outcomes and study designs)

**Table 2:** Mapping of specific clinical outcomes and assessment scales by included source.

Nature of the study	Article title	Clinical outcomes addressed	Key results summary	Author
Randomized Controlled Trial	A physiotherapy group exercise and self management approach to improve physical activity in people with mild-to moderate Parkinson’s disease: a randomized controlled trial	Motor: Functional gait, Daily step count Non-Motor: Behavioral change	Protocol focusing on increasing daily steps and functional gait through behavioral change.	(42)
Systematic Review and Meta-Analysis	A systematic review and meta-analysis on effects of aerobic exercise in people with Parkinson’s disease	Motor: MDS-UPDRS III, TUG, 6MWT, Global motor function	Significant improvements in UPDRS-III, TUG, and 6MWT; aerobic exercise is effective for global motor function.	(5)
Scoping Review	Are movement-based mindful exercises, including Qigong, Tai Chi, and Yoga, beneficial for stroke and Parkinson’s disease? A scoping review	Motor: Motor symptoms, Postural balance Non-Motor: Quality of life	Positive effects on postural balance and motor symptoms; mind-body practices are generally well tolerated.	(32)
Network Meta-Analysis	Comparative efficacy of 24 exercise types in Parkinson’s disease	Motor: Balance, Functional mobility, Gait performance	Network analysis showing dance and multidisciplinary programs as top-ranked interventions for balance.	(14)
Systematic Review and Network Meta-Analysis	Effectiveness of aerobic and resistance training on motor symptoms in Parkinson’s disease: a systematic review and network meta-analysis	Motor: MDS-UPDRS III, Motor symptom severity	Short-period high-intensity resistance training was identified as the most effective strategy for motor symptom reduction.	(26)
Systematic Review and Meta-Analysis	Effects of resistance training on measures of muscular strength in people with Parkinson’s disease: a systematic review and meta-analysis	Motor: Muscular strength (Leg press, Knee extension, 1RM)	Resistance training significantly increased leg press and knee-extension strength compared with control conditions.	(30)
Network Meta-Analysis	Effects of ten different exercise interventions on motor function in Parkinson’s disease patients: a network meta-analysis of randomized controlled trials	Motor: Balance (BBS), Mobility Non-Motor: Quality of life (PDQ-39)	Interactive video games significantly improved balance and quality of life.	(33)
Pilot Study	Feasibility and preliminary efficacy of an online home-based functional exercise program for Parkinson’s disease: a pilot study	Motor: Motor signs (MDS-UPDRS III), Functional performance Non-Motor: Non-motor symptoms (NMSQ), Mood, Fatigue	High compliance with elastic-band and step-box exercises; improvements in motor and non-motor signs.	(41)
Descriptive Study/Report	Functional physical rehabilitation and self-assessment of physical activity in Parkinson’s disease	Motor: Exercise execution accuracy, Daily physical activity	Professional supervision improved accu-racy of exercise execution; rehabilitation increased daily physical activity volume.	(25)
Systematic Review and Bayesian Net-work Meta-Analysis	Optimal dose and type of exercise improve overall balance in adults with Parkinson’s disease: a systematic review and Bayesian network meta-analysis	Motor: Balance, Postural stability, Gait performance	Balance/gait training and multi-component programs were identified as optimal strategies for balance stability.	(34)
Network Meta-Analysis	Parkinson’s disease motor intervention patterns: a network meta-analysis based on patient motor function	Motor: Walking capacity (6MWT distance)	Dance ranked first for improving 6MWT distance; findings support the need for varied interventions.	(2)
Cochrane Network Meta-Analysis	Physical exercise for people with Parkinson’s disease: a systematic review and network meta-analysis	Motor: MDS-UPDRS III, Balance (BBS), Gait	Cochrane review showing that dance and gait/balance training are among the most effective interventions for motor signs.	(8)
Narrative Review	Exergames in neurocognitive disease management in elderly: a narrative review of therapeutic benefits and applications	Motor: Balance, Walking ability Non-Motor: Cognitive function	Exergames are a viable alternative to traditional exercise, significantly improving cognitive functions and balance.	(16)
Systematic Review and Meta-Analysis	The effects of aerobic exercise on quality of life and motor function in patients with Parkinson’s disease: a systematic review and meta-analysis	Motor: Balance (BBS), Motor function (UPDRS-III) Non-Motor: Quality-of-life scores (PDQ-39)	Aerobic exercise significantly improved BBS and overall quality-of-life scores.	(43)
Pilot Study	The feasibility and positive effects of Wuqinxi exercise on cognitive and motor functions: a pilot study	Motor: Motor function (UPDRS-III) Non-Motor: Cognitive function, Attention, Memory	Traditional animal-imitation exercises improved attention, memory, and motor function.	(28)
Systematic Review and Meta-Analysis	The effect of home-based exercise on mtor symptoms, quality of life, and functional performance: a systematic review and meta-analysis	Motor: Motor symptoms (UPDRS-III), Functional performance Non-Motor: Quality of life (PDQ-39)	Remotely supervised exercise was safe and effective; more than three weekly sessions may provide greater motor gains.	(44)
Systematic Review and Network Meta-Analysis	Effectiveness of different exercises in improving postural balance among Parkinson’s disease patients: a systematic re-view and network meta-analysis	Motor: Mobility (TUG), Balance (BBS), Locomotor control (Mini-BESTest)	Exergaming best for TUG; Dance best for BBS; Rhythmical Auditory best for Mini-BESTest.	(31)
Systematic Review	Treadmill training for patients with Parkinson’s disease	Motor: Gait speed, Stride length, Walking distance	Improved gait speed (0.09 m/s) and stride length; small to moderate clinical relevance.	(37)
Systematic Review	Home-based prescribed exercise improves balance-related activities in people with Parkinson’s disease and has benefits similar to center-based exercise: a systematic review	Motor: Balance-related activities (BBS), Walking speed	Improved balance and gait speed; bene-fits similar to equivalent center-based exercise.	(39)
Systematic Review	Dance therapy for Parkinson’s disease: a systematic review	Motor: Tremor, Bradykinesia, Rigidity, Balance (BBS) Non-Motor: Quality of life, Mood	Improved tremor, bradykinesia, and balance; Argentine Tango ranked most effective.	(13)
Systematic Review	The Impact of Physical Activity on Non-Motor Symptoms in Parkinson’s Disease: A Systematic Review	Non-Motor: Global non-motor symptoms (NMSS), Depression (BDI), Apathy, Sleep, Cognition, PDQ-39	Significant positive impact on global non-motor symptoms: depression, apathy, fatigue, sleep, and cognition.	(38)
Systematic Review and Meta-Analysis	Progressive resistance training in Parkinson’s disease: a systematic review and meta-analysis	Motor: Walking speed, Distance (6MWT), Functional mobility (TUG)	No evidence of superiority over other physical training for walking speed, distance, or TUG.	(29)
Survey/Clinical Study	Motivators and Barriers Affecting Exercise in Patients With Parkinson’s Disease	Motor: Physical activity participation Non-Motor: Exercise motivation, Fatigue, Apathy	17% of patients are inactive; non-motor symptoms (fatigue/apathy) are primary barriers, while intrinsic motivation drives exercise.	(12)
Systematic Review and Network Meta-Analysis	Effectiveness of Telemedicine Interventions on Motor and Nonmotor Outcomes in Parkinson Disease: Systematic Review and Network Meta-Analysis	Motor: Total motor symptoms (MDS-UPDRS III) Non-Motor: Depression (HAM-D, GDS), Anxiety	e-Exercise is the most effective remote strategy for total motor symptoms; e-Cognitive interventions best for depression and anxiety.	(27)
Systematic Review and Meta-Analysis	Physiotherapy in Parkinson’s Disease	Motor: MDS-UPDRS III, Balance (BBS), Gait parameters Non-Motor: Quality of life	Meta-analysis confirms significant motor benefits (MDS-UPDRS III), balance (BBS), and gait improvements across modalities.	(1)

Non-motor complications were systematically tracked using the Non-Motor Symptoms Questionnaire/Scale (NMSQ/NMSS), depression/anxiety scales (e.g., BDI, HAM-D, GDS), cognitive screening (MMSE, MoCA), and the Fatigue Severity Scale (FSS). Telemedicine and Digital Health (e-Cognitive): Network meta-analyses mapping remote care by Dou et al. (29) were ranked as the most effective interventions for depression and anxiety. Exergaming and Traditional Mind-Body Practices: Video-game interventions (16) and traditional animal imitation movements (Wuqinxi Qigong) (30) provided distinct cognitive dual-task challenges, leading to significant improvements in executive control, attention, and memory scores. Behavioral Programs and Exercise Barriers: Integrated self-management and behavioral coaching programs (42, 45) effectively addressed non-motor obstacles. Community-based tracking (12) highlighted that while baseline non-motor symptoms like fatigue (FSS) and apathy act as severe barriers causing physical inactivity, targeted behavioral interventions were associated with improvements in exercise participation and intrinsic motivation.

### 3.6 Dropout

Participant dropout represents an important indicator of the feasibility and acceptability of exercise interventions in PD. Across studies, attrition rates were generally below 15% in several well conducted trials (39), suggesting that structured exercise programs are typically well tolerated when appropriately supervised and adapted to participants' capabilities. Higher dropout rates were reported in some inpatient rehabilitation settings, home-based protocols, and technologically mediated interventions (38, 40), indicating that retention may depend substantially on delivery conditions rather than on the exercise modality itself.

Reported examples included 20% dropout in a Zoom-based multi-domain program (38), up to 40% in intensive inpatient rehabilitation protocols (40), approximately 12% in visual-feedback treadmill training (46), 30%–35% in smartphone-delivered yoga or Tai Chi programs (42), 11.5% in high-intensity treadmill training, approximately 6% in remote cycling programs (5), and 18%–20% in progressive resistance training (31). Dance-based interventions showed dropout rates between 10% and 20% across several studies (13). Nonetheless, Karate shows very high adherence among those who complete the course, 87% (45).

#### 3.6.1 Dropout causes

Dropout appeared to be influenced by multiple interacting factors rather than by intervention characteristics alone. Disease related complications such as symptom progression, fatigue, and intercurrent health conditions were frequently reported as reasons for withdrawal and may directly interfere with continued participation in structured exercise programs. Logistical barriers also contributed to attrition, particularly in interventions requiring frequent attendance at rehabilitation facilities. Travel requirements, scheduling constraints, and competing responsibilities may partly explain the higher dropout observed in intensive inpatient programs (40). Logistical constraints such as lack of time, the distance to specialized centers, and financial costs are consistently reported as sig-nificant barriers. In structured trials, logistical issues like family demands and commute difficulties were verified reasons for drop-out (45).

In technology supported interventions, retention appeared to depend not only on accessibility but also on usability and supervision level. Although remote delivery may reduce geographical barriers, difficulties related to digital literacy and reduced real time feedback may still affect adherence (38, 42). Conversely, relatively low dropout rates observed in some supervised and remotely monitored programs suggest that retention improves when interventions are well structured and compatible with daily routines (5, 39). Social engagement may also contribute to sustained participation in dance-based inter-ventions despite moderate variability in attrition rates across studies. The causes of dropout are summarized in Table 3.

**Table 3:** Summary of dropout causes.

Activity focused in source	Article title	Disease stage (H&Y)	Key results summary	Authors
Community-based PA	Motivators and Barriers Affecting Exercise in Patients With Parkinson’s Disease	I–II	17% of patients do not exercise at all; fatigue and apathy are major barriers.	(12)
Telemedicine (Remote)	Effectiveness of Telemedicine Interventions on Motor and Nonmotor Outcomes: Systematic Review and NMA	I–III	Low digital literacy and device complexity remain critical barriers to remote adherence.	(29)
Physiotherapy Trials	Physiotherapy in Parkinson’s disease: A meta-analysis of present treatment modalities.	I–III	High risk of attrition bias: only 22.8% of studies describe Intention-to-Treat analysis.	(1)
Music-Assisted TM	Feasibility of Music-Assisted Treadmill Training (MATT): A Pilot Trial.	II–III	40% dropout rate in the inpatient MATT group due to systemic constraints.	(40)
Karate (Martial Arts)	KICK OUT PD: Feasibility and quality of life in the pilot karate intervention.	I–III	21% dropout rate in the pilot; high adherence (87%) among completers.	(45)
Smartphone-based App	The Efficacy of Tai Chi and Stretching Exercises Based on a Smartphone Application: A Protocol.	I–III	Predicted 30%–35% attrition rate for app-delivered exercises without real-time human feedback.	(42)
Group Exercise and Self-Management	A physiotherapy group exercise and self management approach to improve physical activity in people with mild moderate Parkinson’s disease: a randomized con-trolled trial.	I–III	Significant delays occurred from February 2020 due to the COVID-19 pandemic and team time pressure.	(46)
Online Home-based Exercise (Zoom)	Feasibility and preliminary efficacy of an online home-based functional exercise program for Parkinson’s disease: a pilot study.	I–III	20% attrition rate (12 out of 15 participants completed the study), Device difficulties (handling Apple iPad/Zoom), health-related issues, and conflicts with work commitments.	(38)
Home-based Pre-scribed Exercise	Home-based prescribed exercise improves balance-related activities in people with Parkinson’s disease and has benefits similar to center-based exercise: a systematic review.	I–III	High adherence reported (70%) across the reviewed trials providing data, Unsupervised home exercise is a barrier for patients with cognitive impairment, advanced disease stage, or freezing of gait.	(39)
Dance Therapy	Dance interventions for Parkinson’s disease: A systematic review and meta-analysis.	I–III	High attendance/adherence (frequently exceeding 80%), traditional therapy often fails to sustain interest; dance improves motivation through its social context and group atmosphere.	(13)

## 4 Discussion

This scoping review provides an overview of the exercise modalities most frequently investigated in PD and their reported associations with motor performance, non-motor outcomes, and adherence patterns. The uneven distribution of interventions across the literature appears to reflect methodological feasibility, clinical applicability, and emerging technological innovation. In particular, treadmill-based aerobic training and resistance exercise are widely represented because they can be standardized in terms of intensity and progression and are easily transferable to rehabilitation settings.

Although several network meta-analyses ranked specific exercise modalities more favorably for particular outcomes, these rankings should be interpreted cautiously because they originate from independent evidence syntheses that differed in study populations, intervention protocols, comparators, outcome measures, and statistical methodology. Consequently, the present review does not propose a universal hierarchy of exercise effectiveness but rather summarizes the trends reported across the available literature.

Multi-component programs are similarly prominent due to their ecological validity and their role as comparator interventions in clinical trials, while technology-assisted strate-gies such as exergaming and virtual training environments continue to attract increasing research attention (47, 48). Despite the large number of available studies, comparability across interventions remains limited by substantial heterogeneity in exercise protocols, outcome measures, and reporting of training parameters. In particular, inconsistent application of FITT (Frequency, Intensity, Time and Type) principles restricts the possibility of defining minimal effective exercise doses and limits the translation of research findings into clinical practice (1, 8).

Within these methodological constraints, recurring patterns nevertheless emerge. High-intensity resistance and power training are frequently associated with improvements in global motor symptoms, whereas aerobic and agility-based training show consistent associations with gait speed and endurance outcomes (2, 7, 28). Coordinative and sensorimotor activities such as Tai Chi, dance, and archery-based exercise have also been investigated for their potential role in improving postural control and functional mobility through multidirectional movement, attentional engagement, and rhythmic cueing (13, 29, 43). These benefits may be partly explained by neuro-plastic adaptations and increased neurotrophic factor release associated with repeated motor practice (9). Importantly, exercise intensity and repetition volume appear to influence outcomes at least as strongly as exercise modality alone, although the generalizability of these observations remains limited by sample size variability and disease-stage heterogeneity across studies (28, 49).

Crucially, adherence represents a critical determinant of the effectiveness of exercise interventions in real world settings, suggesting that adherence should be considered a key determinant of intervention success. Evidence suggests that exercise programs should last at least 8 weeks or include at least 30 sessions to induce significant motor benefits (33, 38). Overall, adherence and dropout appear to be influenced by multiple interacting factors rather than by exercise modality alone. Disease-related symptoms, including fatigue, apathy, disease progression, and motor impairment, consistently reduced partic-ipation, whereas individualized exercise prescription, progressive intensity, supervision, and perceived enjoyment were associated with better long-term adherence (5, 12, 14, 40). Technology-assisted interventions increased accessibility and engagement for many participants but also introduced barriers related to digital literacy, device usability, and technology acceptance in some populations (29, 35, 38). Likewise, psychosocial and logistical factors, including transportation, social support, and intrinsic motivation, substantially influenced long-term participation, suggesting that adherence depends more on the interaction between patient, intervention, and context related factors than on the exercise modality itself.

In this context, the “100-Days” program demonstrated that high levels of engagement and zero dropout can be achieved even in remote settings by utilizing a digital platform for real-time guidance and incorporating periodic challenges to sustain participant motivation (41, 44).

Although the extracted data suggest descriptive trends, such as longer retention in supervised or dance-based programs compared to purely technology-based or unmonitored home-based programs, the high heterogeneity of the protocols prevents us from establishing a formal statistical association or a direct causal link between a specific exercise modality and the adherence rate.

Overall, these findings suggest that long-term adherence depends on a complex interaction between patient, intervention, and context-related factors.

Despite the extensive literature on exercise in PD, this scoping review highlights that the evidence remains fragmented and not uniformly distributed across interventions and outcomes. No clearly superior strategy emerges, as benefits appear to vary according to the functional domain addressed; therefore, exercise selection should be tailored to patients' goals, preferences, and abilities. Although optimal training parameters have not yet been definitively established, individualized quantification of physical activity may represent a key factor in achieving the goal of exercise as medicine in PD. Within this perspective, integrating the role of the kinesiologist into a multidisciplinary framework appears essential for designing personalized intervention programs capable of improving both motor and non-motor symptoms while supporting the transfer and maintenance of these benefits in daily life (50).

Importantly, physical activity should not be regarded as an intervention limited to the clinical setting, but rather as a fundamental component of the patient's everyday routine. Individuals should be encouraged to adopt an active lifestyle through structured, progressive, and goal-oriented exercise beyond supervised sessions, thereby promoting autonomy and long-term behavioral change. In this context, the Italian Palestra della Salute model (D.Lgs. 36/2021) represents a practical and scalable framework facilitating the transition from rehabilitation to sustained engagement in physical activity. Overall, integrating structured, supervised, and individualized exercise into daily life may help preserve rehabilitation-related motor improvements, foster long-term participation, and enhance quality of life in people with PD.

Consistent with the objectives of a scoping review, no formal methodological quality assessment or certainty of evidence appraisal was performed. Consequently, the findings should be interpreted as an evidence mapping rather than as a comparative assessment of intervention efficacy.

Most included studies enrolled patients with mild-to-moderate PD (Hoehn & Yahr I–III). Therefore, the applicability of the present findings to advanced Parkinson's disease remains uncertain.

## 5 Conclusions

This scoping review provides an overview of the exercise modalities most frequently investigated in PD and their associations with motor performance, non-motor outcomes, and adherence patterns. Overall, the findings suggest that exercise effects are outcome-specific rather than modality-specific, supporting the need for individualized and goal-oriented prescription. Accordingly, exercise in PD should be viewed as a targeted therapeutic intervention, not merely a generic recommendation. These findings should therefore be interpreted as an evidence map intended to guide future research rather than to establish comparative intervention efficacy.
